# Not All Forms of Independence Are Created Equal: Only Being Independent the “Right Way” Is Associated With Self-Esteem and Life Satisfaction

**DOI:** 10.3389/fpsyg.2020.606354

**Published:** 2021-01-21

**Authors:** Daniela Moza, Smaranda Ioana Lawrie, Laurențiu P. Maricuțoiu, Alin Gavreliuc, Heejung S. Kim

**Affiliations:** ^1^Department of Psychology, West University of Timişoara, Timișoara, Romania; ^2^Department of Psychological and Brain Sciences, University of California Santa Barbara, Santa Barbara, CA, United States

**Keywords:** culture, self-construal, independence, self-esteem, life satisfaction

## Abstract

Past research has found a strong and positive association between the independent self-construal and life satisfaction, mediated through self-esteem, in both individualistic and collectivistic cultures. In Study 1, we collected data from four countries (the United States, Japan, Romania, and Hungary; *N* = 736) and replicated these findings in cultures which have received little attention in past research. In Study 2, we treated independence as a multifaceted construct and further examined its relationship with self-esteem and life satisfaction using samples from the United States and Romania (*N* = 370). Different ways of being independent are associated with self-esteem and life satisfaction in the two cultures, suggesting that it is not independence as a global concept that predicts self-esteem and life satisfaction, but rather, feeling independent in culturally appropriate ways is a signal that one’s way of being fits in and is valued in one’s context.

## Introduction

“The most incredible beauty and the most satisfying way of life come from affirming your own uniqueness.”

Jane Fonda, American actress

“What makes me happy? The fact that I carry my cross by myself.”

Ionut Caragea, Romanian author

A strong and positive association between the independent self-construal and life satisfaction, mediated by self-esteem, has been termed “a pancultural explanation for life satisfaction” (Kwan et al., 1997, p. 1038), because it held true in both individualistic and collectivistic cultures (Kwan et al., 1997; Chang et al., 2011; Duan et al., 2013; Zhang, 2013; Yu et al., 2016). Life satisfaction, self-esteem, and self-construal are individually linked to a wide array of factors, but the idea of researching this particular “pancultural explanation” originated in the findings of an extensive cross-cultural study (Diener and Diener, 1995), which showed a much stronger correlation between self-esteem and life satisfaction in individualistic cultures compared to collectivistic cultures. Subsequent studies established that the independent self-construal is a crucial, individual-level, cultural ingredient that seems to foster self-esteem universally in individuals (Singelis et al., 1999) with further positive implications for life satisfaction across cultures (Kwan et al., 1997). The independent self-construal (or independence) represents the tendency of individuals to define themselves by their unique configuration of internal attributes and to focus on discovering and expressing their distinct potential (Markus and Kitayama, 1991). Independence is more strongly encouraged in individualistic cultures, whereas in collectivistic cultures, interdependence is more strongly encouraged (Markus and Kitayama, 1991); however, members of both types of cultures have both types of self-construals (Singelis, 1994), but only independence is associated with a stronger sense of self-worth and greater life satisfaction in both cultural settings (Kwan et al., 1997). Based on such pancultural findings, independence has been conceptualized and measured as a unidimensional construct and assumed to be experienced and expressed in the same way across all cultures (Singelis, 1994; Gudykunst et al., 1996). Recent approaches to the study of culture find, however, that both independence and interdependence, along with the related cultural dimensions of individualism and collectivism, are more varied than previously assumed and that different cultures favor different ways of being independent or interdependent (see Kusserow, 1999; Vignoles et al., 2016; Campos and Kim, 2017; Kim and Lawrie, 2019).

These new findings raise the question of whether or not there is any cultural diversity in the association between independence, self-esteem, and life satisfaction. If different shades of independence are valued, experienced, and expressed across cultures, it is possible that being independent in ways that are prescribed and valued by one’s culture is associated with increased self-esteem and thus further promotes life satisfaction, but being independent in ways that are not valued by one’s culture is not associated with increased self-esteem. The present research is an attempt to test explicitly whether or not different ways of being independent are more or less linked to self-esteem and, indirectly, to life satisfaction in different cultures.

### The Independence — Life Satisfaction Link

There are two possible theoretical perspectives that can explain the association between independence, self-esteem, and life satisfaction. The first perspective is that independence as a unidimensional construct contributes to self-esteem and life satisfaction across different cultures. This has been the dominant assumption in previous research (Heine et al., 1999). Empirical evidence showed that independence entails the selection of internal (as opposed to social) information in life-satisfaction judgments (Suh et al., 2008), specifically information that promotes and enhances the self (Heine et al., 1999; Lee et al., 2000; Rosopa et al., 2016) and fosters the agentic pursuit (Wojciszke and Bialobrzeska, 2014) of independent hedonic goals (Oishi and Diener, 2001). The self-esteem of highly independent individuals will therefore reflect their perceived success at achieving their independent, agentic, self-promoting, hedonic goals, and consequently, they would be more satisfied with life.

The second theoretical perspective is in line with research findings suggesting that people ascribe higher value to options (e.g., an object or an activity) that are compatible with their goal orientation because they feel “right” due to a high regulatory fit (Higgins et al., 2003; Higgins, 2005). Similarly, fitting in with one’s culture, or experiencing a culture-person fit, has positive implications for self-esteem and well-being (e.g., Leary and Baumeister, 2000; De Leersnyder et al., 2015; Cho et al., 2018). According to this view, even if the overall link between the independent self and life satisfaction is robust across cultures, there may be cultural differences in the “right” way of being independent that lead to increased self-esteem. That is, if different ways of being independent are highlighted and emphasized in different cultures, then being independent in culturally appropriate ways should have positive implications for self-esteem and, indirectly, for life satisfaction. However, being independent in ways that are less emphasized in one’s culture (culturally inappropriate ways) should have few positive implications and possibly even some negative implications for self-esteem and, indirectly, for life satisfaction (Pedrotti and Edwards, 2009; Ryder et al., 2011; De Leersnyder et al., 2014, but see also Ward et al., 2004). Although arguing for the universal importance of cultural fit for self-esteem and life satisfaction, this perspective also allows room for cultural differences in the specific content and definition of independence that can bring about a sense of cultural fit.

### Independence as a Multidimensional Concept

There are different ways to experience and exercise independence, and different cultures may emphasize different ways of being independent. For example, one may feel good about oneself when one stands out and experiences oneself as unique and different; alternatively, one may feel good about oneself when one does not have to rely on anyone else and can take care of oneself.

The classification of cultures based on the individualism-collectivism cultural dimensions (Hofstede et al., 2010) and the independent-interdependent self-construal (Markus and Kitayama, 1991) has provided the theoretical framework for a tremendous amount of research which, in the past several decades, has revealed that psychological processes, including emotions, motivations, and cognitions, are profoundly influenced by culture. Despite the great empirical utility of dividing cultures according to these binary cultural dimensions, this approach has also reduced the complexity and diversity of cultures to an oversimplified contrast between individualistic and collectivistic, independent and interdependent, and East and West. One way that this simple dichotomy between “independence” and “interdependence” has been maintained has been through the widespread use of the Singelis’s self-construal scale (1994), which measures the two dimensions as sperate and distinct constructs. This binary approach has remained the *de-facto* approach despite noteworthy efforts by several researchers to develop more nuanced cultural models of self, such as Gabriel and Gardner (1999), Kashima and Hardie (2000) and Harb and Smith (2008). However, interestingly, most of these models nuanced only interdependence and kept independence as a unitary dimension. A few models did acknowledge different aspects of the autonomy implied by independence (e.g., Singelis et al., 1995; Triandis and Gelfand, 1998; Kagitcibasi, 2005), but, in general, independence has been viewed as a monolithic concept in contrast to a more diversified view of interdependence. At the same time, research conducted looking at the different forms of interdependence demonstrates the value of finer-grained approaches to cultural constructs. Campos and Kim (2017), for example, compared the types of collectivism found in East Asian and Latin American cultures. Although both cultural regions encourage an interdependent view of the self, how interdependence is maintained in relationships is quite different. Similarly, Vignoles et al. (2016) deconstructed both independence and interdependence into their constituent facets and developed a model that distinguishes between different ways of being independent and interdependent across seven different dimensions of functioning (e.g., making decisions, looking after oneself, and communicating with others). The seven dimensions are bipolar in nature, each having an independent pole and an interdependent pole. Initial application of the survey in over 30 countries showed that the seven dimensions did not cluster together into a higher-order dimension of independence and interdependence. Therefore, the conceptualization promoted by Singelis’s measure does not accurately and sufficiently characterize cultural variation in self-construal. Instead, as research by Vignoles et al. (2016) and others suggests, different ways of being independent and interdependent are valued in different cultures. In the current set of studies, we build and expand on this work, testing not only if there are different ways of being independent in different cultures but also if there are psychological implications associated with being or not being independent in ways prescribed by one’s culture.

Whereas previous studies have linked independence, as a unidimensional single factor construct, to self-esteem and life satisfaction, in the current studies, we examine the notion of independence to determine if different aspects of independence are associated with self-esteem and life satisfaction in different cultures. Previous studies found a pancultural explanation, but using a more nuanced approach, we predicted that more cultural differences would emerge. We suggest that it is not independence as a large global concept that predicts self-esteem and, indirectly, life satisfaction, but rather, feeling independent in culturally appropriate ways is a signal that one’s way of being oneself fits in and is valued in one’s context.

### Overview of the Current Research

The current research is made up of two studies. In Study 1, we sought to confirm that the previously found relationship between the single-factor measure of independent self-construal typically used in the literature (i.e., Singelis, 1994), self-esteem, and life satisfaction would hold true in multiple cultures, even cultures that have previously received scant attention in empirical research.

In Study 2, we used Vignoles et al. (2016) model of self-construal to explore further the relationship between independence, self-esteem, and life satisfaction. Using samples from two cultures (the United States and Romania), we examined whether treating independence as a multifaceted construct would reveal considerable variability in the meaning of independence across cultures as well as the implications of different ways of being independent on psychological outcomes such as life satisfaction.

## Study 1: The Relationship Between Unidimensional Independence, Self-Esteem, and Life Satisfaction in Four Cultures

### Introduction

The aim of Study 1 was to test the replicability of previous findings on the link between independence and life satisfaction, mediated by increased self-esteem (Kwan et al., 1997) in cultures that have previously received little empirical attention. To this end, we collected data from three continents and four countries varying on the individualism vs. collectivism index (Hofstede et al., 2010): the United States, 91; Hungary, 80; Japan, 46; and Romania, 30. In addition to the Western individualistic culture (the United States) and the East-Asian collectivistic culture, which have received considerable attention in previous culture research, we therefore included in our study two understudied Eastern European culture – one individualistic (Hungary) and one collectivistic (Romania). Both Hungary and Romania are ex-socialist countries and the socialist regimes strongly promoted collectivism. However, in Hungary, “individualism which was suppressed or kept under control surfaced itself with ‘double strength’ after the political changes when celebrating individualism became the norm (Fülöp et al., 2019, p. 86).” The research reviewed and conducted by Fülöp et al. (2019) suggests that Hungarians, both adults and adolescents, are characterized by high levels of independence. In Romania, instead, the struggle to shake off the legacies of the past regime lead to what Gavreliuc (2011) has termed “autarchic individualism,” a rather ambivalent culture, at the same time individualistic and collectivistic. Mixed results were obtained in various studies using measures of independence, some showing high levels of independence, others low or medium level of independence, irrespective of age (Gavreliuc, 2012; see also David, 2015; Moza, 2018 for reviews). However, David (2015) concluded that a consistent tendency toward independence can be seen among the young and educated (i.e., students). Irrespective of actual levels of independence, independences still has a positive relationship with self-esteem and well-being as has been documented in previous literature. Therefore, we predicted that the relationship between independence and life satisfaction, mediated through self-esteem, would be culturally invariant.

### Materials and Methods

#### Participants and Procedure

Participants were 736 undergraduate students, recruited *via* convenience sampling, from universities in the United States, Romania, Japan, and Hungary. They took part in the study for course credit. The sample consisted of 164 United States (72.6% females; *M_age_* = 20.17, *SD_age_* = 3.58), 199 Hungarian (86.4% females; *M_age_* = 23.83, *SD_age_* = 7.47), 277 Romanian (79.8% females; *M_age_* = 21.83, *SD_age_* = 4.80), and 96 Japanese (44.8% females; *M_age_* = 18.97, *SD_age_* = 1.03) students.

#### Measures

Independent and interdependent self-construals were measured with the popular Singelis (1994) self-construal scale. Fifteen items were used to measure the independent self-construal (e.g., I enjoy being unique and different from others in many respects) and 15 items were used to measure the interdependent self-construal (e.g., “I feel good when I cooperate with others”). Participants rated each item on a seven-point Likert scale ranging from 1 (strongly disagree) to 7 (strongly agree). Higher scores indicated higher levels of independent self-construal (*α* = 0.74 for the United States sample; *α* = 0.72 for the Hungarian sample; *α* = 0.74 for the Romanian sample, and *α* = 0.78 for the Japanese sample) and of interdependent self-construal (*α* = 0.72 the United States sample; *α* = 0.88 for the Hungarian sample; *α* = 0.86 for the Romanian sample, and *α* = 0.71 for the Japanese sample).

Self-esteem was measured with the Rosenberg (1965) Self-Esteem Scale. The scale consists of 10 items (e.g., “I feel that I have a number of good qualities”). Participants rated each item on a four-point Likert scale ranging from 1 (strongly disagree) to 4 (strongly agree). Higher scores indicated higher self-esteem (*α* = 0.91 for the United States sample; *α* = 0.72 for the Hungarian sample; *α* = 0.86 for the Romanian sample, and *α* = 0.85 for the Japanese sample).

Life satisfaction was measured with the Satisfaction with Life Scale (Diener et al., 1985). The scale consists of five items (e.g., I am satisfied with my life). Participants rated each item on a seven-point Likert scale ranging from 1 (strongly disagree) to 7 (strongly agree). Higher scores indicated higher life satisfaction (*α* = 0.90 for the United States sample; *α* = 0.82 for the Hungarian sample; *α* = 0.79 for the Romanian sample, and *α* = 0.85 for the Japanese sample).

Demographic information was obtained on age and gender. Subjective socioeconomic status was measured with the MacArthur pictorial scale (Adler et al., 2000). Participants marked their rung in society compared to others in their environment.

#### Analytic Approach

**Figure 1:**
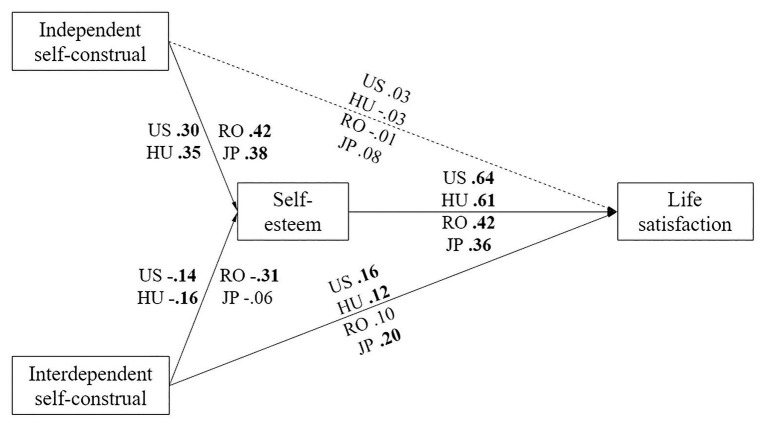
The path model of the relationships between independent and interdependent self-construal, self-esteem, and life satisfaction in all four cultures. In this figure, the values shown are standardized path coefficients; the statistically significant coefficients are shown in bold. Continuous lines represent significant paths in at least one sample (*p* < 0.05), whereas the interrupted line represents non-significant path (*p* > 0.05). US, United States sample; HU, Hungarian sample; RO, Romanian sample; JP, Japanese sample.

Data analysis comprised of four distinct stages: (a) computing descriptive statistics, conducting correlation and ANOVA analyses; (b) performing multi-group SEM to test the mediation model shown in Figure 1; (c) performing bootstrap procedures to test the indirect effects in the mediation model; and (d) testing the invariance of the mediation model as well as *post-hoc* slope comparisons to determine the paths that were significantly different in the four samples.

Main analyses were performed using SEM in Amos 20 (Arbuckle, 2011) and the maximum likelihood estimation method. Gender, age, and subjective socioeconomic status were included as covariates. All variables were identified as observed variables. We decided to include subjective socioeconomic status as a control variable due to its high correlations with both self-esteem (e.g., Twenge and Campbell, 2002) and life satisfaction (e.g., Anderson et al., 2012).

Structural models were evaluated using a constellation of goodness-of-fit indices as recommended by Hu and Bentler (1999), namely the model chi-square, the Comparative Fit Index (CFI – values above 0.95 indicate good fit), the Root Mean Square Error of Approximation (RMSEA – values below 0.06 indicate good fit), and the Standardized Root Mean-square Residual (SRMR – values below 0.08 indicate good fit).

To test the hypothesized mediating effects of self-esteem in the link between self-construals and life satisfaction in a SEM framework, we analyzed the indirect effects of self-construals on life satisfaction using bootstrap functions with 5,000 bootstrap samples and 95% confidence intervals. We used Zhao et al. (2010) mediation typology to distinguish between: (a) complimentary mediation where both the mediated and direct effect exist and point in the same direction, (b) competitive mediation where both mediated and direct effect exist but point in opposite directions, (c) indirect-only mediation where mediation exists but there is no direct effect (d) direct-only non-mediation where only a direct effect exists, and (e) no-effect non-mediation where neither direct nor indirect effect exist.

To test the invariance of the model within the multigroup modeling framework, we constrained the paths of the model to be equal across the four groups and compared this restricted model to a model in which the paths were freely estimated. We examined the change in *χ*^2^ index when cross-group constraints were imposed on the model. In addition, we used ΔCFI as a comparative index, because Δ*χ*^2^ can be affected by sample size (Cheung and Rensvold, 2002). A significant Δ*χ*^2^ and/or a value of ΔCFI smaller than or equal to −0.01 indicates that the fit of the restricted model was significantly worse than the fit of the nonrestricted model, in which case the paths of the model differ significantly across the four groups (Cheung and Rensvold, 2002). The test of the differences between the four groups was performed by using the “Group Differences” tool within the “Stats Tools Package” (Gaskin, 2016). A significant *z*-score indicated significant differences between the groups.

#### Measurement Invariance

Measurement invariance was tested in a structural equation modeling (SEM) framework using confirmatory factor analysis (CFA). Specifically, we tested and established configural, metric, and scalar invariance of each of the three scales. We used the criteria suggested by Chen (2007) to evaluate model fit: ΔCFI smaller than −0.01, ΔRMSEA smaller than 0.015, and ΔSRMR smaller than 0.030. Initial confirmatory analyses yielded small values in the case of discrepancy indices (i.e., CFI and TLI), while fit indices based on residuals (i.e., RMSEA and SRMR) indicated good fit. Based on the conclusions formulated by Kenny et al. (2015), we computed the RMSEA of the null model (i.e., nullRMSEA index) to investigate whether discrepancy indices are adequate for our confirmatory models. Kenny et al. (2015) concluded that discrepancy indices are not valid indicators of fit when the nullRMSEA index is too small (i.e., values below 0.158). The results of the tests of measurement invariance for the three scales in Study 1 are presented in Table 1.

**Table 1:** Tests of measurement invariance for the scales in Study 1.

Scale/model	CFI	RMSEA (90% CI)	SRMR	ΔCFI	ΔRMSEA	ΔSRMR	nullRMSEA
Singelis (1994) Self-Construal Scale							0.134
Configural invariance	ⱡ	0.025 (0.022–0.027)	0.06	ⱡ	--	--	--
Metric invariance	ⱡ	0.027 (0.025–0.029)	0.08	ⱡ	0.021	0.02	--
Scalar invariance	ⱡ	0.028 (0.026–0.030)	0.10	ⱡ	0.022	0.02	--
Rosenberg (1965) Self-Esteem Scale							0.315
Configural invariance	0.968	0.031 (0.024–0.037)	0.03	--	--	--	--
Metric invariance	0.964	0.032 (0.025–0.038)	0.05	0.004	0.001	0.02	--
Scalar invariance	0.954	0.035 (0.029–0.041)	0.08	0.012	0.003	0.03	--
Diener et al. (1985) SWL scale							0.456
Configural invariance	0.994	0.038 (0.021–0.054)	0.01	--	--	--	--
Metric invariance	0.986	0.039 (0.011–0.065)	0.02	0.008	0.010	0.01	--
Scalar invariance	0.975	0.050 (0.036–0.064)	0.05	0.011	0.110	0.03	--

*N* = 736; United States sample *n* = 164; Hungarian sample *n* = 199; Romanian sample *n* = 277; Japanese sample *n* = 96; ⱡ not valid indicators of fit when the nullRMSEA index is too small (i.e., values below 0.158, Kenny et al., 2015).

### Results

Descriptive statistics and the results of one-way ANOVA with *post-hoc* comparisons between the four cultural samples for the variables in the study are presented in Table 2.

**Table 2:** Results of one-way ANOVA with *post-hoc* comparisons between the four cultural groups for the variables in the Study 1 model.

Variable	One-way ANOVA									*Post-hoc* comparisons					
	US		HU		RO		JP			US vs. HU	US vs. RO	US vs. JP	HU vs. RO	HU vs. JP	RO vs. JP
	*M*	*SD*	*M*	*SD*	*M*	*SD*	*M*	*SD*	*F*	*p*					
IND SC	4.91	0.68	4.89	0.64	5.08	0.61	4.26	0.75	37.11^***^	>0.05	<0.05	<0.001	<0.05	<0.001	<0.001
INTER SC	4.88	0.63	4.37	0.79	4.76	0.74	4.62	0.64	18.31^***^	<0.001	>0.05	<0.05	<0.001	<0.05	>0.05
SE	3.76	0.79	3.49	0.73	3.91	0.59	3.20	0.74	30.86^***^	<0.01	>0.05	<0.001	<0.001	=0.01	<0.001
LS	4.78	1.32	4.53	1.18	4.88	1.01	3.94	1.31	16.87^***^	>0.05	>0.05	<0.001	<0.01	=0.001	<0.001

IND SC, independent self-construal; INTER SC, interdependent self-construal; SE, self-esteem; LS, life satisfaction; US, American sample; HU, Hungarian sample; RO, Romanian sample; JP, Japanese sample; M, mean; SD, standard deviation; *p*, *p* value of the *post hoc* comparisons using the Hochberg’s GT2 test for independent self-construal and Games-Howell test for the other three. Levene’s test of homogeneity of variance for independent self-construal was not statistically significant; we therefore used so Hochberg’s GT2 test for *post-hoc* comparisons, because we had unequal groups and equal variances on this variable. For the other three variables, Levene’s test of homogeneity of variance was statistically significant; we considered them as having unequal variances and therefore used the Games-Howell test for *post-hoc* comparisons.

*** *p* < 0.001.

Table 3 presents the bivariate correlations between the variables in each cultural group.

**Table 3:** Bivariate correlations between all variables in the study in all four cultural samples in Study 1.

Variable	1	2	3	4	5	6
American sample (*N* = 164)						
1. Independent self-construal	1					
2. Interdependent self-construal	0.130	1				
3. Self-esteem	0.308^**^	−0.089	1			
4. Life satisfaction	0.271^**^	0.095	0.670^**^	1		
5. Age	0.066	0.199^*^	0.097	0.094	1	
6. Gender	0.140	−0.005	0.108	0.054	−0.157^*^	1
7. SSES	0.072	−0.087	0.152	0.265^**^	−0.105	0.069
Hungarian sample (*N* = 199)						
1. Independent self-construal	1					
2. Interdependent self-construal	−0.068	1				
3. Self-esteem	0.356^**^	−0.184^**^	1			
4. Life satisfaction	0.192^**^	0.027	0.585^**^	1		
5. Age	−0.009	−0.069	0.017	−0.108	1	
6. Gender	0.054	0.052	−0.156^*^	−0.013	−0.031	1
7. SSES	0.052	0.056	0.165^*^	0.250^**^	−0.071	−0.003
Romanian sample (*N* = 277)						
1. Independent self-construal	1					
2. Interdependent self-construal	0.072	1				
3. Self-esteem	0.402^**^	−0.273^**^	1			
4. Life satisfaction	0.151^*^	−0.010	0.387^**^	1		
5. Age	0.101	−0.039	0.064	−0.167^**^	1	
6. Gender	0.001	0.208^**^	−0.005	0.122^*^	−0.065	1
7. SSES	0.019	−0.162^**^	0.083	0.182^*^	−0.001	−0.031
Japanese sample (*N* = 99)						
1. Independent self-construal	1					
2. Interdependent self-construal	−0.261^*^	1				
3. Self-esteem	0.402^**^	−0.177	1			
4. Life satisfaction	0.168^*^	0.113	0.379^**^	1		
5. Age	−0.014	−0.070	0.134	0.152	1	
6. Gender	−0.156	0.182	−0.128	0.013	−0.044	1
7. SSES	−0.014	−0.009	0.033	0.206^**^	0.003	0.009

SSES, subjective socio-economic status.

** *p <* 0.01

* *p <* 0.05.

Based on the results of the preliminary analyses, we initially tested the model presented in Figure 1 without a path from interdependence to life satisfaction because the relationship was not statistically significant in any of the four cultures. The fit indices of this initial model were modest [*χ*^2^(40) = 72.51, *p* = 0.001; CFI = 0.931; SRMR = 0.073, RMSEA = 0.033]. Next, we added an additional path from interdependence to life satisfaction in a second model based on both previous empirical findings (e.g., Kwan et al., 1997; Singelis et al., 1999) and on methodological recommendations (Kline, 2016). This model (see Figure 1) showed improved fit over the initial model [*χ*^2^(40) = 54.07, *p* = 0.068; CFI = 0.970; SRMR = 0.067, RMSEA = 0.022].

The model was not the same across our four cultures. The results of slope comparisons are shown in Table 4.

**Table 4:** Differences in the paths of the model between the four cultural samples in Study 1.

Path in the model	Sample								Statistical comparisons between model paths in the four samples					
	US		HU		RO		JP		US vs. HU	US vs. RO	US vs. JP	HU vs. RO	HU vs. JP	RO vs. JP
	Epc	*p*	Epc	*p*	Epc	*p*	Epc	*p*	*z-score*					
IND SC → SE	0.35	0.000	0.39	0.000	0.41	0.000	0.38	0.000	0.44	0.64	0.24	0.16	0.15	0.30
INTER SC → SE	−0.18	0.051	−0.15	0.012	−0.25	0.000	−0.07	0.543	0.27	−0.74	0.77	−1.43	−0.65	−1.57
SE → LS	1.07	0.000	0.98	0.000	0.71	0.000	0.64	0.000	−0.65	−2.50^**^	−2.11^**^	−1.86^*^	1.66^*^	0.33
INTER SC → LS	0.33	0.005	0.18	0.034	0.14	0.062	0.41	0.030	−1.07	−1.34	0.37	−1.43	−1.13	−1.31
INDSC → LS	0.06	0.616	−0.05	0.636	−0.02	0.866	0.15	0.395	−0.69	−0.49	0.45	0.24	−0.97	−0.83

IND SC, independent self-construal; INTER SC, interdependent self-construal; SE, self-esteem; LS, life satisfaction; US, United States sample; HU, Hungarian sample; RO, Romanian sample; JP, Japanese sample; Epc, estimate path coefficient.

** *p <* 0.05

* *p <* 0.10.

The relationship between self-esteem and life satisfaction was significantly stronger in the United States and Hungarian samples compared to the Romanian and Japanese samples. We found evidence for an indirect-only mediation between independent self-construal, self-esteem, and life satisfaction in all four samples. In other words, independent self-construal has no direct relationship with life satisfaction but only a relationship mediated by self-esteem. In addition, we found evidence for direct-only nonmediation in our United States sample, competitive mediation in our Hungarian sample, indirect-only mediation in our Romanian sample, and no-effect nonmediation in our Japanese sample. The direct, indirect, and total effects of independent and interdependent self-construals on life satisfaction in the four cultural samples are presented in Table 5.

**Table 5:** Direct, indirect, and total effects of independent and interdependent self-construals on life satisfaction in all four cultural samples in Study 1.

Variable	Direct effects		Indirect effects		Total effects	
	*B*(SE) C.I.	*β*(SE) C.I.	*B*(SE) C.I.	*β*(SE) C.I.	*B*(SE) C.I.	*β*(SE) C.I.
American sample						
IND SC	0.06 (0.12) [−0.16, 0.29]	0.03 (0.06) [−0.08, 0.15]	0.37 (0.10)^***^ [0.18, 0.58]	0.19 (0.05)^***^ [0.10, 0.29]	0.43 (0.14)^**^ [0.15, 0.72]	0.22 (0.07)^**^ [0.08, 36]
INTER SC	0.33 (0.12)^**^ [0.09, 0.57]	0.16 (0.06)^**^ [0.05, 0.27]	−0.19 (0.10) [−0.40, 0.01]	−0.09 (0.05) [−0.20, 0.00]	0.14 (0.16) [−0.17, 0.45]	0.07 (0.08) [−0.08, 0.22]
Hungarian sample						
IND SC	−0.05 (0.11) [−0.27, 0.16]	−0.03 (0.06) [−0.15, 0.09]	0.39 (0.08)^***^ [0.24, 0.58]	0.21 (0.04)^***^ [0.13, 0.31]	0.34 (0.12)^**^ [0.09, 0.58]	0.18 (0.07)^**^ [0.05, 0.31]
INTER SC	0.18 (0.09)^*^ [0.01, 0.35]	0.12 (0.06)^*^ [0.01, 0.23]	−0.14 (0.06)^*^ [−0.28, −0.03]	−0.10 (0.04)^*^ [−0.18, −0.02]	0.03 (0.10) [−0.17, 0.24]	0.02 (0.07) [−0.11, 0.16]
Romanian sample						
IND SC	−0.02 (0.10) [−0.21, 0.18]	−0.01 (0.06) [−0.12, 0.11]	0.29 (0.06)^***^ [0.19, 0.42]	0.18 (0.03)^***^ [0.12, 0.25]	0.27 (0.10)^**^ [0.09, 0.46]	0.17 (0.06)^**^ [0.05, 0.27]
INTER SC	0.14 (0.08) [−0.02, 0.30]	0.11 (0.06) [−0.01, 0.22]	−0.18 (0.04)^***^ [−0.26, −0.11]	−0.13 (0.03)^***^ [−0.20, −0.08]	−0.04 (0.08) [−0.20, 0.12]	−0.03 (0.06) [−0.14, 0.09]
Japanese sample						
IND SC	0.15 (0.18) [−0.22, 0.51]	0.09 (0.10) [−0.13, 0.28]	0.24 (0.10)^***^ [0.09, 0.48]	0.14 (0.05)^***^ [0.06, 0.27]	0.39 (0.18)^*^ [0.04, 0.74]	0.22 (0.10)^*^ [0.02, 0.41]
INTER SC	0.41 (0.21)^*^ [0.03, 0.83]	0.20 (0.10)^*^ [0.01, 0.39]	−0.04 (0.08) [−0.23, 0.09]	−0.02 (0.04) [−0.11, 0.05]	0.37 (0.22) [−0.05, 79]	0.18 (0.10) [−0.03, 0.37]

IND SC, independent self-construal; INTER SC, interdependent self-construal; B(SE) and *β*(SE) represent unstandardized (B) and standardized (*β*) coefficients followed by standard errors; C.I. are presented in square brackets and represent 95% bias corrected bootstrap confidence intervals; statistical significance (i.e., a bootstrap approximation obtained by constructing two-sided bias-corrected confidence intervals) is indicated by superscripts.

*** *p <* 0.001

** *p <* 0.01

* *p <* 0.05.

### Discussion

Study 1 results replicated previous findings (Kwan et al., 1997; Chang et al., 2011; Duan et al., 2013; Yu et al., 2016), showing that unidimensional independence and life satisfaction are positively and indirectly related, by self-esteem mediating the relationship. A potential explanation of this mediation mechanism is provided by Markus and Kitayama (1991), who argued that individuals’ own evaluation of their self-worth, which is strongly connected with their life satisfaction, is dependent on the cultural standards encompassed in their self-construal. Our results confirmed the invariance of this mediated relationship in individualistic and collectivistic cultures that have received little attention in past empirical research, such as Hungary and Romania, in addition to well-studied cultures such as the United States and Japan.

## Study 2: Dissecting Independence—An Analysis of Aspects of Independence Associated With Self-Esteem and Life Satisfaction in the United States and Romania

### Introduction

In Study 2, we focused more in depth on two of the countries from Study 1 – the United States and Romania. In Study 1, we established that in both of these cultures, unidimensional independence predicts life satisfaction, and this is partially mediated through self-esteem. Our intent for Study 2 was to see if taking a more nuanced approach and using a new multidimensional measure of independence would illuminate differences between the two cultures.

Previous ethnographic research conducted with European-American parents in different socioeconomic stratums of New York City found that all of the American parents, regardless of family income, embraced an independent view of the self (Kusserow, 1999), but independence meant something very different for lower- and higher-class families. For families of lower SES that had more daily struggle, independence was associated with being tough and self-reliant, but for families of higher SES, independence was associated with developing a unique sense of self. We expected these same types of results at the country level. Indeed, consistent with this theorizing, previous research has shown that in American culture, there is a strong emphasis on self-expression and personal uniqueness (Kim and Markus, 1999; Kim and Sherman, 2007) to the extent that American individualism has been called “expressive individualism” (Bellah et al., 1985). In Romanian culture, although uniqueness is also valued, other characteristics of hard independence, such as self-reliance, consistency, and self-direction are equally valued as uniqueness (Gavreliuc and Ciobotă, 2013). Thus, we predicted that in Romania, a country that is poorer and has dealt with much more upheaval and uncertainty in its recent past (including the collapse of communism and a tumultuous transition to a democracy), aspects of independence that would be valued and associated with self-esteem and life satisfaction would be related to being tough and self-reliant. On the other hand, we expected that in the United States, a relatively wealthier and more stable environment, aspects of independence associated with being unique and standing out would be associated with self-esteem and life satisfaction.

### Materials and Methods

#### Participants and Procedure

Data was collected from a convenience sample of 370 participants. They were 203 Romanian and 167 undergraduate psychology students in the United States who received course credit or extra credit for participating in the study. In the Romanian sample, the mean age was 19.80 years (*SD* = 1.41), 66.5% were females. In the United States sample, 11 participants were excluded from the analyses because they were not fully enculturated in the United States culture (i.e., they were born in another country and immigrated in the United States after they were 5 years old). The mean age of the remaining 156 participants included in the analyses was 18.71 years (*SD* = 1.27), and 64.7% were females.

#### Measures

Independent and interdependent self-construals were measured with the 62-item version of the seven-factor self-construal scale recently developed by Vignoles et al. (2016). Participants indicated the extent to which each of 62 items described them on a nine-point Likert scale ranging from 1 (not at all) to 9 (exactly). The scale includes seven sub-scales reflecting ways of viewing the self as independent of others or interdependent with others with respect to different domains of functioning. Specifically, (1) self-containment vs. connectedness to others with respect to experiencing the self (e.g., “Your happiness is independent from the happiness of your family”; *α* = 0.70 for the United States sample; *α* = 0.75 for the Romanian sample), (2) self-direction vs. receptiveness to influence with respect to making decisions (e.g., “You usually decide on your own actions, rather than follow others’ expectations”; *α* = 0.77 for the United States sample; *α* = 0.76 for the Romanian sample), (3) difference vs. similarity reflects the ways of viewing the self as independent vs. interdependent with respect to defining the self (e.g., “You see yourself as different from most people”; *α* = 0.83 for the United States sample; *α* = 0.76 the Romanian sample), (4) self-reliance vs. dependence on others with respect to looking after oneself (e.g., “You prefer to rely completely on yourself rather than depend on others”; *α* = 0.79 for the United States sample; *α* = 0.76 the Romanian sample), (5) consistency vs. variability with respect to moving between contexts (e.g., “You behave the same way at home and in public”; *α* = 0.89 for the United States sample; *α* = 0.81 for the Romanian sample), (6) self-expression vs. harmony with respect to communicating with others (e.g., “You prefer to say what you are thinking, even if it is inappropriate for the situation”; *α* = 0.78 for the United States sample; *α* = 0.74 for the Romanian sample), and (7) self-interest vs. commitment to others with respect to dealing with conflicting interests (e.g., “Your own success is very important to you, even if it disrupts your friendships”; *α* = 0.70 for the United States sample; *α* = 0.76 for the Romanian sample). Each sub-scale is composed of a certain number of items tapping the independent way of viewing the self and a number of items tapping the interdependent way. Items for both the independent pole and for the interdependent pole of each sub-scale were positively phrased, but conceptual reversals of each other (e.g., consistency: “You behave the same way at home and in public” vs. variability: “You see yourself differently in different social environments”). Items tapping the interdependent self-views were reverse coded. Higher scores on each dimension indicate a higher independent view of the self and lower scores a higher interdependent self-view.

As in Study 1, self-esteem was measured with Rosenberg (1965) Self-Esteem Scale (*α* = 0.90 for the United States sample; *α* = 0.88 for the Romanian sample) and life satisfaction was measured with the Satisfaction with Life Scale (Diener et al., 1985; *α* = 0.88 for the United States sample; *α* = 0.89 for the Romanian sample). Also, as in Study 1, we collected data on age, gender, and subjective socio-economic status.

#### Analytic Approach

The analytic approach was similar to the approach used in Study 1, except for the fact that the model we tested is based on seven-dimensional self-construal and includes only two samples.

#### Measurement Invariance

Measurement invariance was tested in the same way as for the scales in Study 1. The results of the tests of measurement invariance for the three scales in Study 2 are presented in Table 6. Both metric and scalar measurement invariance was achieved, allowing for cross-cultural comparisons using these measures.

**Table 6:** Tests of measurement invariance for the scales in Study 2.

Scale/model	CFI	RMSEA (90% CI)	SRMR	ΔCFI	ΔRMSEA	ΔSRMR	nullRMSEA
Vignoles et al. (2016) Self-Construal Scale							0.127
Configural invariance	ⱡ	0.043 (0.041–0.045)	0.08	ⱡ	--	--	--
Metric invariance	ⱡ	0.044 (0.042–0.045)	0.09	ⱡ	0.001	0.01	--
Scalar invariance	ⱡ	0.044 (0.042–0.046)	0.10	ⱡ	0.000	0.01	--
Rosenberg (1965) Self-Esteem Scale							0.312
Configural invariance	0.960	0.052 (0.039–0.065)	0.06	--	--	--	--
Metric invariance	0.956	0.051 (0.039–0.064)	0.06	0.004	0.001	0.00	--
Scalar invariance	0.957	0.050 (0.038–0.063)	0.07	0.001	0.001	0.01	--
Diener et al. (1985) Satisfaction with Life Scale							0.515
Configural invariance	0.983	0.044 (0.000–0.089)	0.03	--	--	--	--
Metric invariance	0.981	0.040 (0.001–0.084)	0.03	0.004	0.010	0.00	--
Scalar invariance	0.981	0.040 (0.001–0.086)	0.04	0.000	0.110	0.01	--

*N* = 359; United States sample *n* = 156; Romanian sample *n* = 203; ⱡ not valid indicators of fit when the nullRMSEA index is too small (i.e., values below 0.158, Kenny et al., 2015).

### Results

Descriptive statistics and the results of *t*-tests for differences between the United States and Romanian samples for the variables in the study are presented in Table 7.

**Table 7:** Means, SD, and *t*-tests for differences between the United States and Romanian samples for the variables in the Study 2.

Variable	Mean (*SD*)		*t*
	US	RO	
1. Self-containment vs. connectedness to others	4.39 (1.04)	4.55 (1.11)	−1.37
2. Self-direction vs. receptiveness to influence	5.52 (1.15)	6.17 (1.32)	−4.83^***^
3. Difference vs. similarity	5.93 (1.25)	6.55 (1.24)	−4.69^***^
4. Self-reliance vs. dependence on others	5.47 (1.24)	6.35 (1.36)	−6.32^***^
5. Consistency vs. variability	5.10 (1.61)	5.78 (1.55)	−4.04^***^
6. Self-expression vs. harmony	4.74 (1.23)	5.47 (1.33)	−5.32^***^
7. Self-interest vs. commitment to others	4.64 (1.02)	4.75 (1.34)	−0.80
8. Self-esteem	3.01 (0.54)	3.04 (0.53)	−0.47
9. Life satisfaction	4.73 (1.26)	4.67 (1.34)	−0.41

US, American sample; RO, Romanian sample.

*** *p <* 0.001.

Table 8 presents the bivariate correlations between the variables in each sample.

**Table 8:** Bivariate correlations between all variables in Study 2 by each culture.

Variable	1	2	3	4	5	6	7	8	9	10	11	12
1. Cont_Conn	-	0.394^**^	0.007	0.207^**^	0.032	0.201^**^	0.378^**^	0.007	−0.165^*^	−0.096	−0.213^**^	0.039
2. Dir_Rec	0.406^**^	-	0.300^**^	0.460^**^	0.034	0.387^**^	0.276^**^	0.023	−0.162^*^	0.084	−0.141	−0.610
3. Diff_Sim	0.152^*^	0.390^**^	-	0.243^**^	0.304^**^	0.344^**^	−0.087	0.354^**^	0.171^*^	−0.034	0.030	0.088
4. Rel_Dep	0.204^**^	0.523^**^	0.336^**^	-	−0.021	0.072	0.132	−0.058	−0.155	0.101	−0.026	−0.100
5 Cons_Var	0.221^**^	0.163^*^	0.126	0.254^**^	-	0.199^*^	−0.108	0.358^**^	0.212^**^	0.000	0.008	0.133
6. Exp_Har	0.234^**^	0.327^**^	0.332^**^	0.421^**^	0.363^**^	-	0.349^*^	0.208^**^	0.158^*^	−0.046	−0.023	0.033
7. Int_Comm	0.476^**^	0.392^**^	0.107	0.254^**^	0.043	0.260^**^	-	−0.034	−0.090	−0.167^*^	−0.097	0.043
8. Self-esteem	0.100	0.238^**^	0.218^**^	0.352^**^	0.427^**^	0.390^**^	0.153^*^	-	0.706^**^	−0.014	−0.124	0.456^**^
9. Life satisfaction	−0.149^*^	0.051	0.168^*^	0.125	0.285^**^	0.190^**^	−0.142^*^	0.615^**^	-	−0.003	0.041	0.479^**^
10. Age	−0.003	0.159^*^	0.084	0.063	0.032	−0.019	−0.019	0.069	0.033	-	0.121	0.031
11. Gender	−0.158^*^	−0.186^**^	−0.049	0.016	0.042	−0.061	−0.133	0.094	0.128	−0.077	-	−0.098
12. SSES	−0.021	0.005	0.045	0.130	0.153^*^	0.187^**^	0.101	0.350^**^	0.413^**^	0.058	0.052	-

The correlation coefficients of United States sample are presented on the top-right side of the diagonal; the correlation coefficients of Romanian sample are shown on the down-left side of the diagonal; Diff_Sim, Difference vs. Similarity; Cont_Conn, Self-containment vs. Connection to others; Dir_Rec, Self-direction vs. Receptiveness to influence; Rel_Dep, Self-reliance vs. Dependence on others; Exp_Har, Self-expression vs. Harmony; Int_Comm, Self-interest vs. Commitment to others; Cons_Var, Consistency vs. Variability; SSES, Subjective Socio-economic Status; Cronbach’s alpha coefficients for the United States sample are presented on the left side and for the Romanian sample, on the right side.

** *p <* 0.01

* *p <* 0.05.

**Figure 2:**
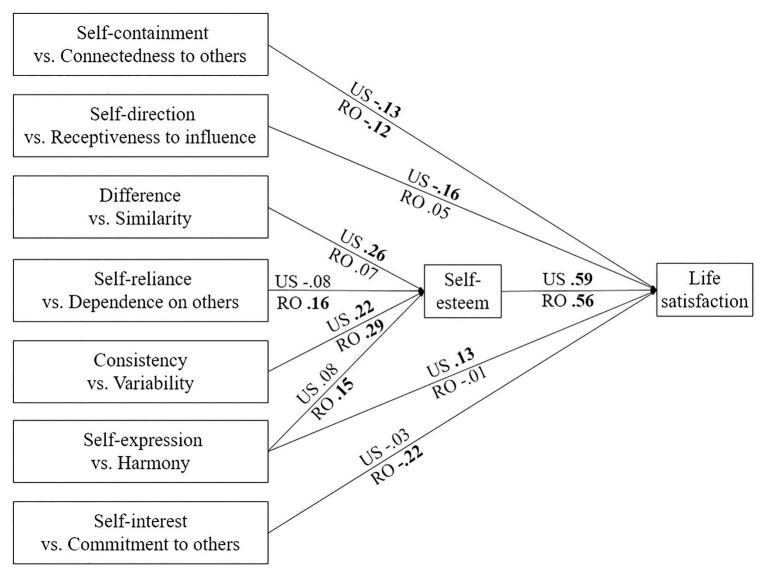
Path model of the relationships between self-construal dimensions, self-esteem, and life satisfaction. In this figure, the values shown are standardized path coefficients. The paths that were not statistically significant in at least one sample are not showed; the statistically significant coefficients are shown in bold; US, United States sample; RO, Romanian sample.

We initially built a path model, which included all the paths from the self-construal dimensions to self-esteem and to life satisfaction for which the correlations were statistically significant in at least one sample. This initial model also included all the significant correlations between the different self-construal dimensions. The model showed an excellent fit [*χ*^2^ = 30.74, *df* = 22, *p* = 101; CFI = 0.990; SRMR = 0.041; RMSEA = 0.033 CI 10% (0.000, 0.059)]; however, the direct paths from the self-construal dimensions of self-direction vs. receptiveness to influence and self-interest vs. commitment to others and self-esteem, and between the self-construal dimension of difference vs. similarity and life satisfaction were non-significant in both cultural groups and were thus removed in the subsequent model. The modified model (Figure 2) had an excellent fit, slightly improved over the initial model [*χ*^2^ = 39.52, *df* = 32, *p* = 0.169; CFI = 0.991; SRMR = 0.040; RMSEA = 0.026 CI 10% (0.000, 0.049)]. As predicted, the model was different across cultures.

The differences in the paths of the model between the two cultural samples are shown in Figure 2 (see also Table 9).

**Table 9:** Differences in the paths of the model between the two cultural samples in Study 2.

Path in the model	Romanian sample		U.S. sample		*z*
	Epc	*p*	Epc	*p*	
Difference vs. similarity → self-esteem	0.108	0.000	0.029	0.276	−1.921^*^
Self-reliance vs. dependence on others → self-esteem	−0.035	0.233	0.063	0.013	2.530^**^
Consistency vs. variability → self-esteem	0.072	0.002	0.098	0.000	0.826
Self-expression vs. harmony → self-esteem	0.032	0.292	0.059	0.028	0.687
Self-esteem → life satisfaction	1.375	0.000	1.432	0.000	0.282
Self-containment vs. connectedness to others → life satisfaction	−0.159	0.027	−0.141	0.045	0.180
Self-direction vs. receptiveness to influence → life satisfaction	−0.166	0.012	0.049	0.408	2.427^**^
Self-expression vs. harmony → life satisfaction	0.127	0.009	−0.008	0.885	−1.614
Self-interest vs. commitment to others → life satisfaction	−0.038	0.610	−0.213	0.000	−1.849^*^

Epc, estimate path coefficient; *z*, the Z (Fisher) test.

** *p <* 0.05

* *p <* 0.10.

Next, we tested the indirect effects of the self-construal dimensions on life satisfaction through self-esteem (indirect-only mediation, where mediation exists but there is no direct effect, Zhao et al., 2010). In the United States sample, two self-construal dimensions had statistically significant positive indirect effects on life satisfaction, namely difference vs. similarity [*B* = 0.15(0.049), 95% CI (0.06, 0.25), *p* < 0.01; *β* = 0.15(0.049), 95% CI (0.06, 0.25), *p* < 0.01] and consistency vs. variability (*B* = 0.10(0.039), 95% CI [0.03, 0.18], *p* < 0.01; *β* = 0.13(0.049), 95% CI [0.04, 0.33], *p* < 0.01). In the Romanian sample, there were three self-construal dimensions that had positive indirect effects on life satisfaction, namely self-reliance vs. dependence on others (*B* = 0.09(0.039), 95% CI [0.01, 0.17], *p* < 0.05; *β* = 0.09(0.040), 95% CI [0.01, 0.17], *p* < 0.05), consistency vs. variability (*B* = 0.14 (0.037), 95% CI [0.07, 0.22], *p* < 0.001; *β* = 0.16(0.042), 95% CI [0.09, 0.25], *p* < 0.001), and self-expression vs. harmony (*B* = 0.09 (0.041), 95% CI [0.00, 0.17], *p* < 0.05; *β* = 0.09(0.041), 95% CI [0.00, 0.17], *p* < 0.05).

We then tested direct-only nonmediation, where only a direct effect exists between self-construal dimensions and life satisfaction, in each cultural sample. As shown in Figure 2, four self-construal dimensions predicted life satisfaction directly. Only one of these dimensions predicted life satisfaction positively, and only in the United States sample, and that is self-expression vs. harmony [*B* = 0.13(0.057), 95% CI (0.01, 0.24), *p* < 0.05; *β* = 0.13(0.058), 95% CI (0.01, 0.24), *p* < 0.05]. The other predictors were negative. This means that a higher level of the independent pole of a self-construal dimension was associated with lower life satisfaction, whereas a higher level of the interdependent pole of the same dimension was associated with higher life satisfaction. There was only one dimension that predicted life satisfaction similarly, and negatively, in both cultures, namely self-containment vs. connectedness to others [the United States sample: *B* = −0.16 (0.082), 95% CI (−0.32, −0.01), *p* < 0.05; *β* = −0.13(0.069), 95% CI (−0.27, −0.00), *p* < 0.05; Romanian sample: *B* = −0.14 (0.076), 95% CI (−0.30, −0.00), *p* < 0.05; *β* = −0.12(0.062), 95% CI [−0.24, −0.00], *p* < 0.05). The dimension self-direction vs. receptiveness to influence predicted life satisfaction negatively only in the United States sample [*B* = −0.17 (0.067), 95% CI (−0.30, −0.03), *p* < 0.05; *β* = −0.16(0.063), 95% CI (−0.28, −0.03), *p* < 0.05]. The dimension self-interest vs. commitment to others predicted life satisfaction negatively only in the Romanian sample [*B* = −0.21 (0.056), 95% CI (−0.31, −0.09), *p* = 0.001; *β* = −0.22(0.059), 95% CI (−0.32, −0.09), *p* = 0.001].

### Discussion

Study 2 provides initial evidence suggesting that there is more cultural diversity in the link between independence, self-esteem, and life satisfaction than previously thought. Interestingly, although the commonly used unidimensional measure of independence (i.e., Singelis, 1994) was a positive predictor of self-esteem and, indirectly, of life satisfaction in both Romania and the United States in Study 1, when using a more nuanced approach in Study 2, we found two sets of significant differences between the two cultural samples in the specific ways of being independent that are associated with self-esteem and life satisfaction. First, in the relationship between self-construal and self-esteem, there were significant differences at the level of two self-construal dimensions. The self-construal dimension linked to self-esteem in the United States sample, but not in the Romanian sample was difference vs. similarity, whereas the dimension linked to self-esteem in the Romanian sample, but not in the United States sample was self-reliance vs. dependence on others. These results are in line with previous research showing that for Americans, discovering and expressing personal uniqueness is a normative cultural task (Kim and Markus, 1999; Kim and Sherman, 2007). For Romanians, instead, being self-reliant is normative, especially among young and educated adults (Gavreliuc and Ciobotă, 2013) who increasingly tend to take their fate into their own hands in order to create a better life for themselves compared to their parents. For instance, Sandu (2010) has argued that the participation of Romanians in the massive wave of migration for better work opportunities has led to increased self-esteem among those who have managed to be self-reliant and improve their standards of living.

Second, in the relationship between self-construal and life satisfaction, there were differences at the level of two other self-construal dimensions. Specifically, the self-construal dimension linked to life satisfaction in the United States sample, but not in the Romanian sample was self-direction vs. receptiveness to influence, whereas the dimension linked to life satisfaction in the Romanian sample, but not in the United States sample, was self-interest vs. commitment to others. These relationships were negative, meaning that high self-direction in the United States and high self-interest in Romania were related to lower life satisfaction, whereas high receptiveness to influence in the United States and high commitment to others in Romania were related to higher life satisfaction. These were rather unexpected findings and not in line with previous findings for either American or Romanian cultures. However, previous research using the unidimensional model of self-construal found direct positive relationships between the interdependent self-construal and life satisfaction in both collectivistic and individualistic cultures (e.g., Hong Kong – Kwan et al., 1997; United States – Ross and Murdock, 2014). Our results suggest that in the same way that different ways of being independent are related to life satisfaction indirectly, through increased self-esteem, different ways of being interdependent can also be related directly to life satisfaction in different cultures.

## General Discussion

### Conclusion

Previous empirical work has found a positive association between the independent self-construal and life satisfaction, mediated through self-esteem in many different cultures. Based on this research, the assumption in the literature has long been that the relationship between independence and life satisfaction is mediated by self-esteem and is universally the same and cross-culturally invariant. Employing a commonly used unidimensional measure of independence (i.e., Singelis, 1994) in Study 1, we tested this assumption and replicated the findings in four different cultures, including Romania and Hungary, which have received scant attention in past research. In Study 2, however, using a more nuanced approach including Vignoles et al. (2016) newly developed seven-dimension self-construal model, we expected to find much more cultural variability in the association between independence, self-esteem, and life satisfaction. The results were in line with these expectations. In contrast with the culturally invariant model in Study 1, the model in Study 2 showed significant cultural differences in the relationship between two self-construal dimensions (i.e., difference vs. similarity – significant only in the United States sample; self-reliance vs. dependence on others – significant only in the Romanian sample) and self-esteem and in the relationship between two other self-construal dimensions and life satisfaction (i.e., self-direction vs. receptiveness to influence – significant only in the United States sample; self-interest vs. commitment to others – significant only in the Romanian sample).

Our two studies yielded three main sets of relevant findings. First, when measured unidimensionally, independence is linked to life satisfaction through self-esteem in both individualistic and collectivistic cultures. This finding suggests that there is a universal mechanism by which independence promotes life satisfaction by enhancing individuals’ sense of self-esteem. The second set of findings form out research, however, suggest that there are both common (e.g., consistency vs. variability in both the United States and Romania) and distinct ways of being independent that are valued across different cultures and associated with self-esteem and life satisfaction (e.g., difference vs. similarity in United States and self-reliance vs. dependence on others in Romania). A potential explanation for the relationships between self-construal dimensions and self-esteem that are common among different cultures is that they might be based on universal human motivations. For example, the finding that individuals who tend to behave in accordance with their self-concept and strive to keep their self-views intact (i.e., increased consistency) also have better evaluations of their self-worth (i.e., increased self-esteem) might be a universal rather than a culturally-specific association, because it underlies a basic human motivation (Elliott, 1986; Suh, 2002). For instance, Church et al. (2014) found that consistency was positively related to well-being in both individualistic and collectivistic cultures. Another potential explanation is the existence of common cultural values and norms regarding appropriate ways of being independent (Suh et al., 2008) in the cultures in which the same relationships between ways of being independent and self-esteem hold true. As a consequence, individuals possessing culturally appropriate ways of being independent would experience higher cultural fit, with positive effects on their sense of self-worth and, indirectly, on their life satisfaction (Suh et al., 2008; Pedrotti and Edwards, 2009; Ryder et al., 2011; De Leersnyder et al., 2014). This same explanation can be applied to the findings that showed cultural differences in the relationship between self-construal and self-esteem, such as the positive relationship between difference vs. similarity and self-esteem in our United States sample and between self-reliance vs. dependence on others in our Romanian sample. Americans who view themselves as unique and different from others would experience a higher cultural fit and their sense of self-worth would be higher compared to Americans who view themselves as more similar to others. Similarly, Romanians who view themselves as more self-reliant would experience a higher cultural fit and would have a higher self-esteem compared to Romanians who view themselves as dependent on others. These findings are in line with research by Becker et al. (2014) which found that individuals across 20 cultural groups derived self-esteem mostly on the basis of values consistent with the priorities of their culture and less based on values they endorsed personally.

Finally, the third set of findings showed that in addition to the indirect relationship between independence and life satisfaction, mediated through self-esteem, there are also direct, mainly negative, relationships between ways of being independent and life satisfaction. These direct relationships may also be different in different cultures (e.g., self-direction in the United States and self-interest in Romania). A potential explanation for these findings is that each culture has relationship norms (Suh et al., 2008; Kim and Lawrie, 2019) that individuals have to follow in order to act in culturally appropriate ways (e.g., being receptive to the influence of others or being committed to others). Individuals who act according to inner motivations that are contrary to these cultural norms for good relationships with others would be rejected by others and experience a diminished sense of belonging with detrimental consequences on life satisfaction (Baumeister and Leary, 1995).

In addition to the main findings which were the result of testing the proposed mediation models in the two studies, there were also some unexpected findings resulting from the comparison of the specific cultural samples included in the current studies. For example, the Romanian sample scored higher on life satisfaction compared to the other four cultural samples under investigation. This was surprising given that Romania typically has some of the lowest subjective well-being scores on international surveys [e.g., Eurofound (2013) reported that Romania lies second from the bottom out of 27 EU countries on overall well-being]. However, our results are in line with those obtained by Krys et al. (2020), where the scores of the Romanian sample were exceeded only by four of the 50 cultures included in the study. One explanation for these striking results could be that in both our sample, and the sample included in the Krys et al. (2020) study, were composed of university students, whereas the national samples include participants of all ages. Recent research by Lawrie et al. (2020) showed a negative relationship between age and life satisfaction in Romania. Therefore, it is possible that younger samples might experience a reference effect such that they are comparing themselves to considerably unhappier older individuals. Another surprising and unexpected finding was that the American sample in Study 1 had the highest interdependence scores among our four cultural samples. Similarly, the United States sample in Study 2 scored higher on the interdependent poles of six of the seven dimensions of self-construal. A potential explanation is offered by Markus (2017), who suggests that high interdependence can be found in Americans who are working-class and/or people of color. Our American student samples were mixed in terms of both race/ethnicity and social class so it is possible that their high scores on interdependence are due to the specific characteristics of the sample under investigation.

Overall, our findings suggest that by conceptualizing independence as a broad global concept, much of the subtle ways in which culture impacts psychological processes may be ignored. It appears that being independent in the ways prescribed by one’s culture, that is, being independent the right way, signals that one belongs and fits in with one’s cultural group, and this cultural fit may be one of the keys to self-esteem and life satisfaction. The current studies are the first to show not only that independence varies across the two cultures under investigation (i.e., the United States and Romania), but that there are also different psychological implications associated with being independent in different ways.

### Limitations and Suggestions for Future Research

There are some limitations in the current studies that could be addressed in future research. First, both studies relied on student samples from a limited number of cultures. Yet different ways of being independent are likely to be associated with self-esteem and life satisfaction in different non-student samples. As previous research suggested, generations are specific types of cultures (Moss and Martins, 2014); therefore, our results might not be the generalized to samples of older adults. Building on the findings of this research regarding the cultural variability in these associations, future studies might therefore test them in both student and nonstudent samples from a wider array of cultural regions. Second, the current research is cross-sectional in nature and, although the mediation models we tested suggest a specific direction of the associations (i.e., from self-construal dimensions to self-esteem and, further, to life satisfaction), only longitudinal designs such as the one employed by Moza et al. (2019) could inform correctly on their directionality. Moreover, future studies might test experimentally the causality of the relationships in the model, informing potential interventions to boost life satisfaction in people from various cultures.

## Data Availability Statement

The raw data supporting the conclusions of this article will be made available by the authors, without undue reservation.

## Ethics Statement

The studies involving human participants were reviewed and approved by ethics committees of West University of Timișoara and of University of California, Santa Barbara, respectively. The patients/participants provided their written informed consent to participate in this study.

## Author Contributions

DM and SL share equal first authorship of this article. All authors jointly developed the ideas presented in this article. DM, SL, AG, and HK designed the studies and collected the data. Data were analyzed by DM, SL, and LM. DM, SL, HK, and LM drafted the article. All authors provided critical revisions, and all authors approved the final version of the manuscript for submission.

### Conflict of Interest

The authors declare that the research was conducted in the absence of any commercial or financial relationships that could be construed as a potential conflict of interest.
